# An Empirical Study on Patients’ Acceptance of Physician-Patient Interaction in Online Health Communities

**DOI:** 10.3390/ijerph16245084

**Published:** 2019-12-12

**Authors:** Xinyi Lu, Runtong Zhang, Xiaomin Zhu

**Affiliations:** 1 School of Economics and Management, Beijing Jiaotong University, No. 3 Shangyuancun, Haidian District, Beijing 100044, China; xinyilu@bjtu.edu.cn (X.L.); rtzhang@bjtu.edu.cn (R.Z.); 2 School of Mechanical, Electronic and Control Engineering, Beijing Jiaotong University, No. 3 Shangyuancun, Haidian District, Beijing 100044, China

**Keywords:** online health communities (OHCs), unified theory of acceptance and use of technology (UTAUT), physician-patient interaction, health information, structural equation modelling

## Abstract

In China, the utilization of medical resources is contentious, and a large of hospitals are seriously congested because of the huge population and uneven distribution of medical resources. Online health communities (OHCs) provide patients with platforms to interact with physicians and to get professional suggestions and emotional support. This study adopted the unified theory of acceptance and use of technology to identify factors influencing patients’ behavioral intention and usage behavior when interacting with physicians in OHCs. An investigation involving 378 valid responses was conducted through several Chinese OHCs to collect data. Confirmatory factor analysis and structural equation modelling were utilized to test hypotheses. Both the reliability and validity of the scales were acceptable. All five hypotheses were supported, and behavioral intention played a significant mediating role between independent variables and dependent variables. This study clarified the mechanism by which performance expectancy, effort expectancy, social influence and attitude toward using technology affect usage behavior through the mediation of behavioral intention in OHCs. These findings suggest that OHCs can change the actions of websites such as adopting some incentives to promote patients’ intention of interaction. Physicians should understand patients’ actual attitudes toward OHCs and try to guide patients in their interactions, improving the quality of physician–patient interaction.

## 1. Introduction

In China, the utilization of medical resources is contentious, and most hospitals are seriously congested because of the huge population and uneven distribution of medical resources. Online health communities (OHCs) can alleviate this situation to a certain extent. OHCs provide people with platforms to obtain and exchange health-related information [1]. To specify, patients can seek health information and communicate with physicians to acquire specialized suggestions and emotional support through OHCs [2,3,4], so they can diagnose themselves with some simple diseases and protect their privacy when they have a stigmatized illness [5,6]. Physicians can publish health-related articles, share health information, and answer patients’ questions to help patients and improve their social reputation [7].

In OHCs, patients can flexibly ask for help at any time, and physicians can answer patients’ questions when they are unoccupied, improving the utilization of physicians’ time [3,5,6]. However, it may be difficult for patients to express their conditions accurately only by words, and the symptoms of some diseases are similar, which may make it difficult for physicians to determine patients’ illnesses. In addition, the time for physicians to see questions, understand patients’ conditions, and answer patients may be long without real-time interaction, which may delay patients’ diagnoses. Hence, what do patients think of physicians in OHCs, and will they interact with physicians in OHCs or not?

Venkatesh et al. [8] developed the unified theory of acceptance and use of technology (UTAUT) based on the eight most widely used theories in information system acceptance [9]. UTAUT is a well-established theoretical framework [10] to explain the intention to use a technology [11] and can explain 70% of the variance, which is better than other information and communication technology (ICT) adoption models [12]. Therefore, this study used UTAUT to examine the factors influencing patients’ acceptance of physician–patient interaction in OHCs, which is a highly subjective question.

## 2. Research Model and Hypotheses

### 2.1. Online Health Communities

As virtual communities, online health communities (OHCs) can be divided into three categories from the aspects of users: (1) one which is only for patients to discuss health-related topics, and share and exchange information; (2) one which is for patients to ask for help from physicians and for physicians to publish health-related information; and (3) one which is only for physicians to exchange and share their professional knowledge. This study focused on the second type.

Patients can create posts about their conditions and questions in OHCs, and other users may answer these questions and provide advice. Patients can also choose a physician to carry out one-to-one and not face-to-face interaction, and their privacy can be protected [13,14,15]. Therefore, patients can diagnose themselves with simple symptoms under the guidance of physicians in OHCs, which is beneficial to alleviate the congestion in hospitals and increase resource utilization [3,7,16]. In addition, OHCs are platforms for emotional communication [17].

However, as an Internet channel, OHCs have several downsides. First, the quality of information may not be guaranteed [17]. Because of the features of zero gatekeeping and zero-cost publishing [18], everyone can make posts in OHCs without ensuring information quality. Although the identities of physicians in OHCs have to be certified, patients cannot confirm whether they are obtaining answers from a real physician. Another problem is that physicians cannot accurately understand patients’ conditions without face-to-face interaction, which may cause mistakes and reduce patient satisfaction. In consideration of OHCs’ advantages and unstoppable development, it is important to identify patients’ attitudes toward interaction with physicians in OHCs. However, studies related to the driven factors of patients interacting with physicians are limited.

### 2.2. Unified Theory of Acceptance and Use of Technology

The unified theory of acceptance and use of technology (UTAUT) was developed by Venkatesh et al. [8] based on the eight most widely used models, including four determinants—performance expectancy (PE), effort expectancy (EE), social influence (SI), and facilitating conditions (FCs)—which can explain 70% of the variance and better explain the determinants of behavioral intentions than the existing eight models [12]. Mäntymäki and Salo [19] used UTAUT to examine the impact of motivation, social influence, perceived network size, user interface, and facilitating conditions on the intention to purchase in social virtual worlds. Dermentzi and Papagiannidis [20] mainly discussed the driven factors influencing the UK public’s intention to use online technologies as a tool in academia. In terms of the application of healthcare, Sun and Lu [21] applied UTAUT to identify the determinants of people’s use intentions of healthcare websites. Veer et al. [11] analyzed the factors influencing older people’s behavioral intention and usage behavior of e-Health.

### 2.3. Physician—Patient Interaction

Interaction is an essential element of the physician–patient relationship, the importance of which is second only to family relationships [22]. As Heritage and Maynard [23] summarized, patients’ positive attitudes in the interaction with physicians are useful to improving health outcomes [24], especially for patients with chronic diseases. To promote patients’ active attitudes, physicians should pay attention to three things: improving the physician–patient relationship, promoting information exchange, and enabling patients to be involved in decision-making [25,26]. However, physicians may be less likely to understand their patients’ desires accurately because of the differences and complexities of patients’ demands. In addition, the interaction is often physician-centered because patients always lack experience, which makes patients hold the view that they may not obtain enough information [26]. Without face-to-face communication, it may be less likely to be physician-centered. Patients have adequate time to consider physicians’ opinions and suggestions, and patients who use OHCs may have higher health literacy and experience more interactions with physicians.

### 2.4. Research Hypotheses

**Figure 1:**
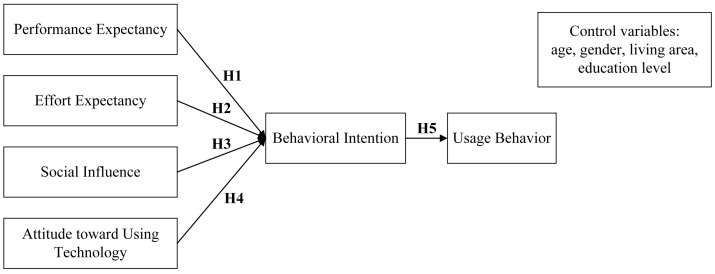
Research model.

Based on UTAUT, this study developed a research model including four independent variables, one mediator, one dependent variable, and four control variables, as shown in Figure 1. Since users have basic skills to use OHCs, this research model removed the facilitating condition (FC) [21] and included attitude toward using technology (AUT) as an independent variable [21]. Age, gender, living area, and education level were taken into account in this study as control variables to identify effects of demographic variables and adjust results.

To specify, PE refers to the services of physicians in the physician–patient interaction in OHCs, which can meet patients’ requirements of improving life quality and health conditions. EE refers to patients’ perceived ease of interacting with physicians in OHCs. SI refers to the impact of other people’s feelings, views, and behaviors on the behavioral intention of patients interacting with physicians in OHCs. AUT refers to patients’ overall affective reactions to interaction with physicians in OHCs. Behavioral intention refers to the possibility of patients thinking they will interact with physicians in OHCs. Usage behavior refers to the actual interaction with physicians in OHCs at a particular time [8,21]. Therefore, we proposed the following hypotheses:

**Hypothesis 1:**  *Performance expectancy (PE) has a positive impact on behavioral intention (BI)*.

**Hypothesis 2:**  *Effort expectancy (EE) has a positive impact on behavioral intention (BI)*.

**Hypothesis 3:**  *Social influence (SI) has a positive impact on behavioral intention (BI)*.

**Hypothesis 4:**  *Attitude toward using technology (AUT) has a positive impact on behavioral intention (BI)*.

**Hypothesis 5:**  *Behavioral intention (BI) has a positive impact on usage behavior (UB)*.

## 3. Materials and Methods

### 3.1. Instrument Development

This study used the multiple-item scale with a 5-point Likert-type response format that ranged from “strongly disagree” to “strongly agree” to measure each construct covering six variables in the research model, as shown in Table 1.

Prior to the investigation, we had to translate the English questionnaire into Chinese, since this survey would be conducted in China. Drawing lessons from the previous translation processes [27,28], we first recruited native Chinese speakers who had at least a Master’s degree, fluently spoke English, and were skilled in scientific research translation to translate our scales into Chinese, considering the cross-cultural adaptation. Then, we invited individuals from different backgrounds including age, gender, living area, education level, and employment to read the Chinese version and help modify the scales to ensure clarity, comprehensibility, conciseness, readability, and the approximate completion time.

### 3.2. Data Collection and Respondent Profile

Our subjects were Chinese individuals who had ever interacted with physicians in OHCs. From April to May 2018, the investigation was anonymously conducted through several Chinese OHCs, and we stated that the privacy of participants was protected and their informed consent was secured. Ethics approval (case reference number: BJTUSEM201803011) was obtained from the Ethics Committee of School of Economics and Management, Beijing Jiaotong University in China in which the study was undertaken. We received 453 responses, and 378 of them were valid. Table 2 shows the demographics of the research sample, 228 (60.32%) participants were 20–40 years old, 206 (54.50%) were females, and 319 (58.46%) had at least a Bachelor’s degree. People who use Internet channels (i.e., OHCs) are younger, female, and highly educated [1,29,30], and Yan et al. [1] found that age, gender, and education level did not have any significant effect on knowledge sharing in OHCs. Therefore, the sample could meet our requirements.

## 4. Results

### 4.1. Data Analysis

The SPSS 22.0 and AMOS 22.0 (Armonk, New York, NY, USA) were used to analyze data and test hypotheses. Cronbach’s alpha values of PE, EE, SI, AUT, BI, and UB were 0.734, 0.805, 0.709, 0.733, 0.803, and 0.765, respectively, and were bigger than 0.700, so the reliability was acceptable [31]. The Kaiser–Meyer–Olkin (KMO) value was 0.960, so the collected data were suitable for factor analysis [32,33,34,35].

Examining the discriminant validity of constructs, we established six nested confirmatory factor analytic models based on the research model and compared their fits to the data [36]. Model 1 was a six-factor model treating each of the variables as separate factors; model 2 was a five-factor model treating performance expectancy and effort expectancy as the first factor, treating social influence as the second factor, treating attitude toward using technology as the third factor, treating behavioral intention as the fourth factor, and treating usage behavior as the fifth factor; model 3 was a four-factor model treating performance expectancy, effort expectancy, and social influence as the first factor, treating attitude toward using technology as the second factor, treating behavioral intention as the third factor, and treating usage behavior as the fourth factor; model 4 was a three-factor model treating performance expectancy, effort expectancy, social influence, and attitude toward using technology as the first factor, treating behavioral intention as the second factor, and treating usage behavior as the third factor; model 5 was a two-factor model treating performance expectancy, effort expectancy, social influence, attitude toward using technology, and behavioral intention as the first factor, and treating usage behavior as the second factor; and model 6 was a one-factor model treating all six factors as one factor. Table 3 shows the fit indices of nested models, and model 1 was the best model in terms of fit to the data. Therefore, all six constructs were distinct from each other, so the discriminant validity was acceptable, and the indicators loaded onto their intended latent variables.

### 4.2. Hypothesis Testing

Considering the incorporation of the measurement error and detection of effects, structural equation modelling (SEM) was adopted to test the hypotheses [37]. Results indicated that patients of different ages, genders, living areas, and education levels did not have any significant difference in behavioral intention and usage behavior regarding physician–patient interaction in OHCs.

**Figure 2:**
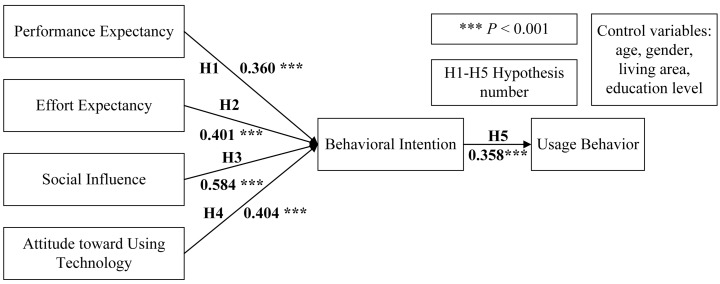
Research model with path coefficients.

In terms of the hypotheses, as shown in Figure 2, all effects were significant, and the path coefficients of H1, H2, H3, H4, and H5 were 0.360, 0.401, 0.584, 0.404, and 0.358, respectively. Therefore, all five hypotheses were supported. Specifically, performance expectancy, effort expectancy, social influence and attitude toward using technology had direct impacts on patients’ behavioral intention regarding physician–patient interaction and had indirect impacts on their behavior of interacting with physicians in OHCs. We adopted the bootstrapping method (*n* = 5000, 95% confidence interval (CI)) to examine the mediation of behavioral intention, and the estimated effects are presented in Table 4. Because all effects were significant, behavioral intention played a significant mediating role between (1) performance expectancy and usage behavior, (2) effort expectancy and usage behavior, (3) social influence and usage behavior, and (4) attitude toward using technology and usage behavior.

## 5. Discussion

### 5.1. Principal Results

This study examines factors influencing patients’ behavioral intention and usage behavior of patients interacting with physicians in OHCs based on UTAUT, and it makes several theoretical and practical contributions to the future study of physician–patient interaction in OHCs and UTAUT. First, we used UTAUT to identify the factors influencing patients’ usage behavior regarding their interaction with physicians. We clarified the mechanism by which performance expectancy, effort expectancy, social influence and attitude toward using technology affect usage behavior through the mediation of behavioral intention in OHCs. The behavioral intention has a positive impact on usage behavior, so OHCs should increase the behavioral intention of patients to interact with physicians, such as adopting some incentives to promote patients’ intention of interaction.

Second, social influence plays the strongest role in directly influencing patients’ behavioral intention and indirectly influencing the usage behavior of interacting with physicians in OHCs. As previous studies have reported [38,39,40], social influence has a strong and positive effect on individuals’ behavior. Therefore, social influence can encourage patients to interact with physicians in OHCs to an extent. When patients intend to choose physicians to interact in OHCs, they often refer to physicians’ reputation in terms of social influence. When patients trust their physicians, they are likely to introduce these physicians to their relatives and friends. Therefore, physicians can increase the frequency of interacting with patients in OHCs and strengthen their reputation to improve their social influence.

Third, effort expectancy has a positive impact on the behavioral intentions of patients interacting with physicians in OHCs. Regardless of the fact that most participants were highly educated, they may be less experienced in interacting with physicians in OHCs, which shows that the physician–patient interaction in OHCs has not achieved the standard of being simple, friendly, and easy to use. Therefore, OHCs need to modify the actions of websites related to physician–patient interaction, and physicians should focus on guidance in the process of interaction to improve the user experience and motivate patients’ behavioral intention.

Fourth, attitude toward using technology positively influences the behavioral intentions regarding physician–patient interaction in OHCs. Attitude toward using technology is defined as “an individual’s overall affective reaction to using a system” [8]. However, the use of physician–patient interaction in OHCs may differ from other technologies. In China, physicians sometimes require their patients to use OHCs and communicate in terms of follow-up treatments, which may promote patients who take negative attitudes toward OHCs to use OHCs. Patients interacting with physicians in OHCs may not mean they largely accept it. This finding suggests that physicians should understand patients’ actual attitudes toward OHCs and try to introduce the benefits of OHCs to patients to promote patients’ active interaction with physicians through OHCs.

Lastly, the results show that the effect of performance expectancy on behavioral intention is the smallest among the effects of independent variables on behavioral intention. Although patients may consider physician–patient interaction in OHCs to be less useful to improve their health conditions than offline treatments, performance expectancy plays an important role in promoting patients to interact with physicians in OHCs. Therefore, both OHCs and physicians can work to improve the quality and effects of physician–patient interaction in OHCs to inspire patients’ intention and behavior regarding interacting with physicians in OHCs.

### 5.2. Limitations and Potentials

Several limitations and potentials of this study must be considered. First, our subjects were Chinese individuals, and the investigation was conducted through several Chinese OHCs. Despite the fact that the characteristics of our sample were consistent with OHC users, we did not consider the accurate user structure of OHCs in China, nor did we consider the Chinese population, and the number of respondents was relatively small. Second, the dimensions of the phenomenon in China are given neither in China nor in other areas. Given that the development of healthcare and OHCs in China is special, future studies can compare the similarities and differences between China and other regions. Third, we measured the variables only once, which maybe failed to detect the dynamic changes in patients’ views. Fourth, diseases can be included as a control variable into the research model in future studies, exploring the differences of attitudes toward physician–patient interaction in OHCs between patients with chronic diseases and others. Fifth, the sample in this study is young, female, highly educated, and living in urban areas, which is consistent with the characteristics of current OHC users. In the future, OHCs users may cover all age spectra, education levels, and living areas, and some new and interesting results could be obtained through repeating this study. Sixth, it may be conceivable to include some moderators into the research model, such as patients’ regulatory focus.

## 6. Conclusions

This study aims to discuss patients’ acceptance of physician–patient interaction in OHCs, employing the research model established based on UTAUT. The results reveal that performance expectancy, effort expectancy, social influence and attitude toward using technology have positive effects on behavioral intention, and behavioral intention has a positive effect on behavior. These findings suggest that (1) OHCs should change the actions of websites related to physician–patient interaction and adopt some incentives to promote patients’ intention of interaction; (2) physicians should understand patients’ actual attitudes toward OHCs, try to guide patients in the interaction, and improve the quality and effects of physician–patient interaction; (3) physicians can increase the frequency of interacting with patients in OHCs and strengthen their reputation to improve their social influence.

## Figures and Tables

**Table 1:** Measurement instrument.

Constructs	Items
Performance expectancy (PE) [8,21]	1. I would find the physician–patient interaction in an online health community useful for my health. 2. Conducting an interaction with physicians in an online health community can improve my life quality. 3. Conducting an interaction with physicians in an online health community increases my ability for self-care. 4. Conducting an interaction with physicians in an online health community makes me become healthier.
Effort expectancy (EE) [8,21]	1. Physician–patient interaction in an online health community would be clear and understandable. 2. It would be easy for me to become skillful at physician–patient interaction in an online health community. 3. I would find the physician–patient interaction in an online health community easy to maintain. 4. Learning to conduct the physician–patient interaction in an online health community is easy for me.
Social influence (SI) [8,21]	1. People who influence my behavior think that I should conduct an interaction with physicians in ab online health community. 2. People who are important to me think that I should conduct an interaction with physicians in an online health community. 3. I will feel uneasy if my friends conduct an interaction with physicians in an online health community but I do not.
Attitude toward using technology (AUT) [8,21]	1. Physician–patient interaction in an online health community is a bad/good thing. 2. Physician–patient interaction in an online health community makes healthcare more easily to understand. 3. Physician–patient interaction in an online health community is useful. 4. I like to conduct interactions with physicians in an online health community.
Usage behavior (UB) [8,21]	1. Recently, I plan to conduct an interaction with physicians in ab online health community. 2. I’m willing to conduct an interaction with physicians in ab online health community if available. 3. I think I will be willing to conduct an interaction with physicians in an online health community if I have known some. 4. I will conduct an interaction with physicians in an online health community in the future.

**Table 2:** Demographics of sample.

Characteristic		Number	Percentage
(1) Age	<20	18	4.76%
	21–29	119	31.48%
	30–39	109	28.84%
	40–49	81	21.43%
	50–59	45	11.90%
	60 and above	6	1.59%
(2) Gender	Male	172	45.50%
	Female	206	54.50%
(3) Living area	Urban	247	65.34%
	Rural	131	34.66%
(4) Education level	Junior middle school and below	9	2.38%
	High school	50	13.23%
	Junior college	98	25.93%
	Bachelor’s degree	183	48.41%
	Master’s degree	33	8.73%
	Doctor’s degree	5	1.32%

**Table 3:** Comparison of measurement models in confirmatory factor analysis.

Model Factors	Fit Indices ^1^						
	χ^2^	*df*	χ^2^/*df*	RMSEA	CFI	IFI	TLI
Model 1 (six factors)	378.828	215	1.762	0.045	0.958	0.959	0.951
Model 2 (five factors)	397.607	220	1.807	0.046	0.955	0.955	0.948
Model 3 (four factors)	445.161	224	1.987	0.051	0.944	0.944	0.936
Model 4 (three factors)	448.448	227	1.976	0.051	0.944	0.944	0.937
Model 5 (two factors)	488.376	229	2.133	0.055	0.934	0.934	0.927
Model 6 (one factor)	489.045	230	2.126	0.055	0.934	0.934	0.927

^1^*χ*^2^ = Pearson’s Chi-square; *df* = degrees of freedom; RMSEA= root mean square error of approximation; CFI= comparative fit index; IFI = incremental fit index; TLI = Tucker–Lewis index.

**Table 4:** Estimates of effects by bootstrapping method.

Effects ^1^	Path coefficients (SD)	*P*
PE → BI	0.351 (0.128)	0.003
EE → BI	0.392 (0.092)	0.000
SI → BI	0.570 (0.115)	0.000
AUT → BI	0.400 (0.112)	0.001
BI → UB	0.165 (0.045)	0.000
PE → UB	0.058 (0.024)	0.002
EE → UB	0.065 (0.021)	0.000
SI → UB	0.094 (0.033)	0.000
AUT → UB	0.066 (0.025)	0.000

^1^ PE = performance expectancy; EE = effort expectancy; SI = social influence; AUT = attitude toward using technology; BI = behavioral intention; UB = usage behavior.
